# A Positive Correlation between Steroid Injections and Cuff Tendon Tears: A Cohort Study Using a Clinical Database

**DOI:** 10.3390/ijerph19084520

**Published:** 2022-04-08

**Authors:** Ching-Yueh Lin, Shih-Chung Huang, Shiow-Jyu Tzou, Chun-Hao Yin, Jin-Shuen Chen, Yao-Shen Chen, Shin-Tsu Chang

**Affiliations:** 1 Department of Physical Medicine and Rehabilitation, Kaohsiung Armed Forces General Hospital, Kaohsiung 802301, Taiwan; linchingyueh@gmail.com; 2 Department of Physical Medicine and Rehabilitation, Tri-Service General Hospital, School of Medicine, National Defense Medical Center, Taipei 114202, Taiwan; 3 Division of Cardiology, Department of Internal Medicine, Kaohsiung Armed Forces General Hospital, Kaohsiung 802301, Taiwan; sghung@gmail.com; 4 Teaching and Researching Center, Kaohsiung Armed Forces General Hospital, Kaohsiung 802301, Taiwan; jyu0120@gmail.com; 5 Institute of Medical Science and Technology, National Sun Yat-sen University, Kaohsiung 804201, Taiwan; 6 Division of Cardiology, Department of Internal Medicine, Tri-Service General Hospital, National Defense Medical Center, Taipei 114202, Taiwan; 7 Department of Medical Education and Research, Kaohsiung Veterans General Hospital, Kaohsiung 813414, Taiwan; mo521141@gmail.com; 8 Institute of Health Care Management, National Sun Yat-sen University, Kaohsiung 804201, Taiwan; 9 Department of Administration, Kaohsiung Veterans General Hospital, Kaohsiung 813414, Taiwan; dgschen@vghks.gov.tw (J.-S.C.); yschen@vghks.gov.tw (Y.-S.C.); 10 Department of Physical Medicine and Rehabilitation, Kaohsiung Veterans General Hospital, Kaohsiung 813414, Taiwan

**Keywords:** cuff tendon tear, shoulder disease, steroid injection

## Abstract

This cohort study aimed to investigate the association between steroid injections for shoulder diseases and the increased incidence of cuff tendon tears. The Kaohsiung Veterans General Hospital clinical database was used in this study. Patients were enrolled using the corresponding diagnostic codes for shoulder diseases. Patients who received steroid injections were included in the case group, and those without steroid injections were included in the control group. The outcome measure was the occurrence of cuff tendon tears during the study period. Adjusted hazard ratios for outcomes were calculated using Cox regression analysis adjusted for sex, age, and comorbidities. Of the 1025 patients with shoulder disease, 205 were in the case group and 820 were in the control group. The incidence of cuff tendon tears was 9.8% in patients who received steroid injections (*p* < 0.001). The adjusted hazard ratios for steroid injections, smoking, and chronic liver disease were 7.44 (*p* < 0.001), 2.40 (*p* = 0.046), 3.25 (*p* = 0.007), respectively. Steroid injections on the shoulder were associated with a raised risk of cuff tendon tears by 7.44 times compared to non-injection. The incidence of cuff tendon tears increased by 3.25 times with concurrent chronic liver disease and by 2.4 times with smoking.

## 1. Introduction

Shoulder pain is common during a clinical visit to a primary care physician, making up 1.2% of general practice visits and ranking only after back and neck discomfort. Shoulder pain accounts for 6.9–34% of the entire population and 21% of those older than 70 years [1]. Shoulder pain can also be attributed to diverse reasons: from structural aspects such as rotator cuff tendon, bursa, bone, and joint; functional aspects such as impingement syndrome, as well as adhesive capsulitis; to a broader entity such as referred pain from visceral or adjacent organs. Improperly treated shoulder pain can cause physical and mental morbidities and profoundly increase the socioeconomic burden. Hence, precise diagnosis and treatment planning are essential, leading to satisfactory outcomes. The therapeutic effects of manual therapy and exercise, low-level laser therapy or electrotherapy, botulinum toxin injections, and arthroscopic distension with corticosteroids have been shown in previous studies but with little evidence [2,3,4,5]. The advantages of local steroids, such as rapid pain-mitigation and anti-inflammatory effects, make them a widely adopted option other than physiotherapy for different musculoskeletal complaints. In a series of Cochrane reviews, steroid injections provided substantial clinical effects in terms of pain reduction and functional regain and were superior to physiotherapy and systemic NSAIDs administration in terms of efficacy [1,6,7]. It also proficiently dealt with postoperative shoulder stiffness without increasing the tear size or retear rate [8,9,10,11]. However, these beneficial effects are not long-lasting [1,6,7].

Recently, attention has been focused on the deleterious effects of local steroids on the tendons. Animal studies have shown that local steroids can degrade collagen, decrease fibroblast proliferation, increase inflammation and cytotoxicity, and interfere with the normal biomechanics in tendons [12,13,14]. Recently, adverse effects related to steroid injections have been demonstrated in human tendon studies, including collagen degradation [15], fibroblast aging [16], and NMDAR1-related excitotoxicity and impaired collagen healing in human tendons [17]. Other than steroids alone, other contributors were involved in tendon rupture to varying degrees, including age, dominant hand, smoking, osteoporosis, hypertension, and gout [18,19,20,21,22,23]. Hence, this study aimed to investigate whether steroid injection is associated with an increased incidence of rotator cuff tendon tears (RCT) and to explore the possible risk factors for RCT formation.

## 2. Materials and Methods

This study was conducted using the clinical database of the Kaohsiung Veterans General Hospital (KSVGH), which is based in southern Taiwan, and encompasses 815,990 consecutive outpatients from January 2013 to December 2019. Demographic data of the study population, including sex, age, smoking status, alcohol use, and medical records, were accessed from the registry. Diagnoses and comorbidities were defined using the corresponding codes of the International Classification of Diseases, Ninth and Tenth Revision, Clinical Modification (ICD-9-CM and ICD-10-CM) (Table S1). The study design was approved by the KSVGH Committee on Human Research (KSVGH20-CT-16), which also waived the requirement for informed consent because of the retrospective observational design of the study and the lack of increased health risk in the patient. This study was conducted in accordance with the Declaration of Helsinki (1964).

### 2.1. Inclusion and Exclusion Criteria 

**Figure 1:**
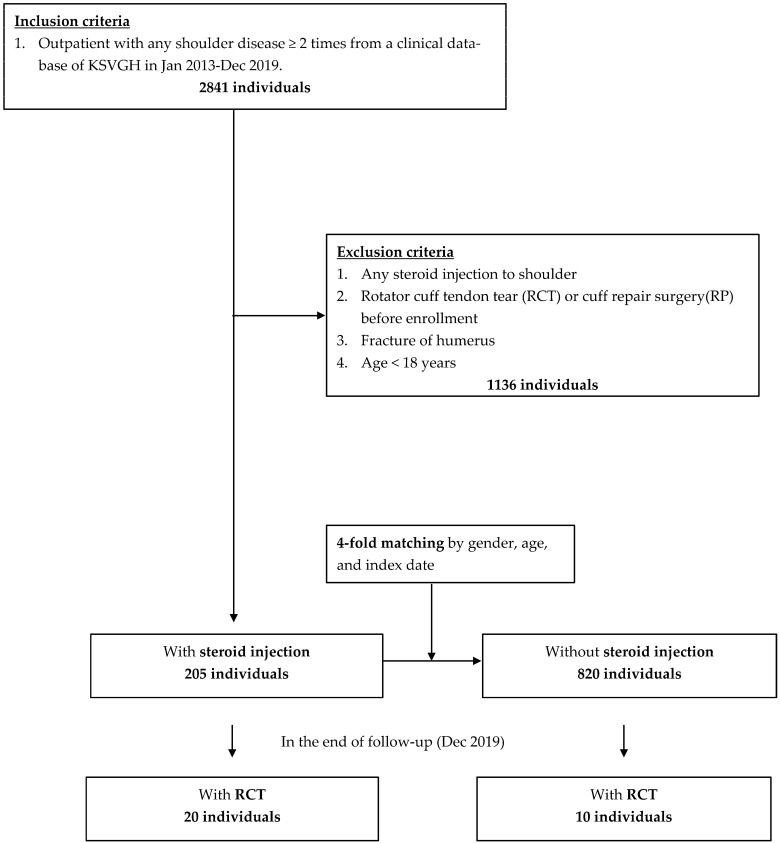
Flowchart.

As shown in Figure 1, patients with shoulder diseases, those who had a corresponding diagnostic code in the first three ranks as outpatients at least twice, were included in this study (Table S1). Exclusion criteria included previous steroid injections to the shoulder, humerus fracture, the occurrence of RCT or cuff tendon repair (RP) before the index date, and age of <18 years. Patients who received steroid injections for shoulder diseases were assigned to the case group, whereas those who did not were assigned to the control group. The index date was either the date of the first administration of steroid injections in the case group or the first date of enrollment in the control group. Group matching was performed at a ratio of 1:4 for the case and control groups, balanced by age, sex, and index date.

### 2.2. Intervention

Steroid injections for patients with shoulder diseases were recognized as having a diagnostic code in the first three ranks, procedure code, and drug code at the same outpatient visit. Procedure codes for intra-articular injection, tendon injection, and trigger point injection were applied. A drug code for the steroid triamcinolone was used (Table S1).

### 2.3. Outcome

The outcome of the study was the occurrence of RCT or its equivalent, with RP at any time during the study period. The corresponding diagnostic and procedure codes are listed in Table S1. 

### 2.4. Comorbidities

Comorbidities, including thyroid disorder, diabetes, gout, depression, hypertension, ischemic heart disease, chronic liver disease, chronic kidney disease, connective tissue disease, and osteoporosis, were used to analyze the risk of RCT (Table S1).

### 2.5. Statistical Analysis

SAS software (SAS System for Windows, version 9.2; SAS Institute, Cary, NC, USA) and SPSS statistical software 22.0.0 (IBM Corp., Armonk, NY, USA) were used to perform all statistical analyses. Descriptive statistics were used to analyze the baseline demographic data and distribution of each variable among the study population. Categorical variables were described as proportions and compared using the χ^2^ analysis with Fisher’s exact correction, whereas continuous variables were expressed as mean ± SD and analyzed using an independent *t*-test. Overall survival rates were calculated using the Kaplan–Meier method, and differences in survival were determined using the log-rank test. The purposeful selection process begins with a univariate Cox regression of the collected parameters. Any variable with a significant univariate test at a *p* < 0.10 was selected as a candidate for the multivariate analysis [24]. Stepwise Cox regression with backward selection was used for the overall analysis of death in the multivariate model. The hazard ratios (HR) and their 95% confidence intervals (CIs) from Cox regression analyses were used to estimate the relative risk.

## 3. Results

A total of 1025 patients with shoulder diseases were enrolled from the KSVGH clinical database between 2013 and 2019. Of these, 205 patients treated with steroid injections were enrolled in the case group, while 820 patients who were never treated with steroid injections were included in the control group (Figure 1). They were followed up for an average of 49 months (data not shown). As shown in Table 1, the case number of shoulder diseases in females and males was 614 (60%) and 411 (40%), with a slight female predominance. The mean age of those with shoulder diseases was 59.4 years old, while that of patients who ultimately developed RCT was 62.2 years old. 

The incidence of RCT in our study was 2.9% (30/1025), with 9.8% (20/205) in the case group and 1.2% (10/820) in the control group. The mean duration from case enrollment to the time of onset of RCT was 39 ± 22 months, and there was no difference in the time of onset between the two groups. Patients with RCT were more likely to have a smoking habit and concurrent diseases, such as gout, hypertension, or chronic liver disease. Of the 205 patients in the case group, 156 (76%), 33 (16%), and 16 (8%) patients received one, two, and three or more times the steroid injections, respectively. There were also no significant outcome differences according to the number of injections.

**Figure 2:**
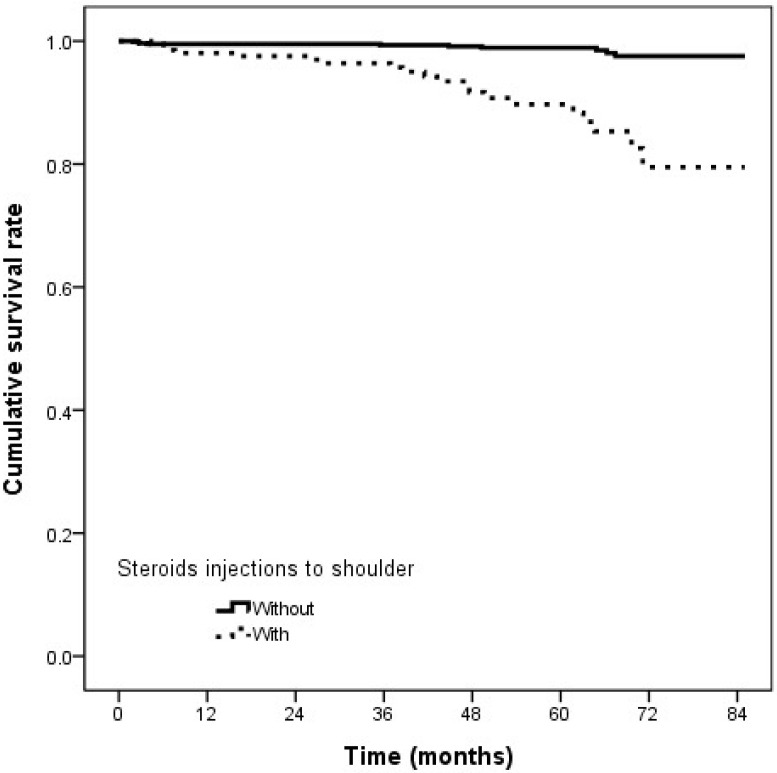
Cumulative survival curve of outcomes.

In the univariate Cox regression analysis (Table 2), steroid injections, smoking, gout, hypertension, and chronic liver disease were significantly associated with RCT. In the stepwise Cox regression with backward analysis (Table 3), only steroid injections, smoking, and chronic liver disease remained significantly correlated with RCT. The adjusted HR for steroid injections, smoking, and chronic liver disease were 7.44 (CI 3.45–16.00; *p* < 0.001), 2.40 (CI 1.02–5.66; *p* = 0.046), 3.25 (CI 1.38–7.63; *p* = 0.007), respectively. The cumulative survival curves of the outcomes are shown in Figure 2.

## 4. Discussion

To our knowledge, this is the first retrospective cohort study to survey the incidence of RCT after steroid injections in patients with shoulder diseases, as sourced from a clinical database of a medical center in southern Taiwan that is equivalent to a tertiary care hospital. In our study, the overall incidence rate of RCT was 2.9%, with incidence rates of 9.8% and 1.2% for those who received steroid injections for shoulder diseases and those who did not, respectively. We found that patients will have a higher HR of 7.44 to develop RCT after steroid injections for a shoulder condition within a mean duration of 39 months. Furthermore, patients who have chronic liver disease and cigarette use will also incline to a higher HR of 3.25 and 2.4 to suffer from RCT in the future. 

There has been continuous efforts on studying the deleterious effects of steroids. In animal models, steroid injections cause a decrease in tensile force [12] and adversely interfere with biomechanics [13] in rabbit Achilles tendons. In addition, reduced tendon durability, bone quality, and integrity of the osteotendinous junction [14], as well as increased risk of inflammation, necrosis, and fragmentation of collagen bundles by repeated injections [25,26], have been noted in rat rotator cuff tendons. A comprehensive review by Dean et al. [27] clearly summarized that steroids have potentially harmful effects on tendons in three dimensions. Histologically, it interrupts the organization and increases the necrosis of collagen, decreases the proliferation and viability of fibroblasts, and increases inflammation, cytotoxicity, and adhesion. At the cellular level, it decreases collagen synthesis, increases collagen apoptosis, and dysregulates enzymes and cytokines. Mechanically, this might reduce the mechanical properties of the tendons. Only two in vivo human tendon studies were included in the review [27]; Lee and Ling verified steroid-induced collagen disorganization and necrosis in the human Achilles tendon [15], and Poulsen et al. showed that fibroblast senescence was noted in the steroid-injected human cuff tendon [16]. At the molecular level, steroids play a significant role in the modulation of gene signaling to interfere with the transcription complex, thus altering the inflammatory cascade [28]. Dean et al. later found an increased NMDAR1 level in steroid-injected human tendons and that NMDAR may play a key role in mediating cell damage and death [17]. Despite this, there are differing opinions. Steroid injections to normal Achilles in rabbits were protective by downregulating metalloproteinases at 48 h [29]. Adverse effects, such as alteration in collagen composition and extracellular matrix by steroid injections in rat cuff tendons, were temporary (7-day) and self-limited [30]. Collagen expression did not change following steroid injections in injured rat cuff tendons [31]. The inconsistent results may be explained by the variable methodology and heterogeneity in these studies. 

There is still little evidence regarding the incidence of RCT after steroid injections in human cuff tendons. In a retrospective case-controlled study, Bhatia et al. did not find any significant correlation between the occurrence of RCT and doses of steroid injections using magnetic resonance imaging for evaluation [32]. Two other studies used large clinical databases to evaluate the risk of tendon tears after repair surgery when preoperative steroid injections were administered. They had consistent conclusions with some nuances: Traven et al. [33] indicated steroid injections within 6 months before surgery will increase the risk of revision surgery, while Desai et al. [34] indicated that two or more steroid injections within 1 year before surgery bring a higher risk of tendon tear to patients. The softening of cuff tendons by steroid injections reported by Watson [35] can explain why patients with preoperative steroid injections were prone to re-tear after surgical fixation [33,34], and it also showed a dose-dependent trend [35]. Ramirez et al. reported an incidence rate of 17% for full-thickness tendon tears in patients receiving subacromial steroid injections at 12 weeks [36]. This study design had better diagnostic accuracy by using ultrasound examination before and after the intervention, avoiding the inclusion of asymptomatic RCT initially, thus preventing the overestimation of the number of RCT. 

The incidence rate of RCT in our study was 2.9% for all participants but surged to 9.8% when participants had received steroid injections. Regarding the prevalence of RCT in the general population, less than 5% of complete cuff tears in more than 500 cadavers were reported by Neer [37] and 6.7% in 268 cadavers were reported by Yamanaka [38]. Lehman et al. divided their sample into two groups by age and found that 6% of cadavers under 60 years old and 30% of those over 60 years old already had RCT, with a higher incidence in the older group [39]. The incidence rate of RCT development after steroid injections was 9.8% in our study, lower than the 17% reported by a prospective study by Ramirez et al. [36], but still higher than that in the general population [37,38,39]. The overall incidence rate of RCT (2.9%) being lower in our population compared to other studies [37,38,39] may account for the lower incidence rate of RCT (9.8%) in our study group. The mean age at RCT was 62.2 years in our study, consistent with previous studies [36,37,39]. A slight female tendency to develop shoulder diseases and RCT was noted, which may be explained by differences in shoulder activities and constitutional factors between sexes. We did not find any significant difference in outcomes with respect to the number of steroid injections, similar to the findings of another study [34]. In our study, we determined a mean duration of 39 months from steroid injection to the onset of RCT formation. 

Smoking was the main risk factor for RCT formation in our study. The causal relationship between smoking and RCT formation has been verified in a systemic review [23] in a dose-dependent and time-dependent manner [18]. Interestingly, chronic liver disease was also a major risk factor for RCT development in our study, with an adjusted HR of 3.25. Currently, no study has established a direct correlation between cuff tendon tears and liver disease. Ma et al. [40] confirmed that chronic liver disease associated with higher serum gamma-globulins and lower immune complexes correlated well with joint derangement and arthritis, and one plausible explanation was that chronic liver disease-related anemia exacerbated inadequate oxygen and nutrition supply to tendons. Yang’s review [41] explained why musculoskeletal disorders prevail following liver disease. Osteoporosis is mainly caused by the upregulation of the receptor activator of the nuclear factor kappa (RANK)-RANK ligand (RANKL)-osteoprotegerin system, increased inflammatory cytokines, and inhibited bone formation by accumulated bilirubin and sclerostin and decreased insulin-like growth factor-1 in liver disease. However, sarcopenia is associated with obesity and insulin resistance in non-alcoholic fatty liver disease, while hyperammonemia, deficiency of certain branched amino acids, and hypergonadism account for sarcopenia in liver cirrhosis. Based on current knowledge, the crosstalk between osteopenia [19,31,42] and sarcopenia [35] fundamentally affects cuff tendon health. Dougherty’s review [43] further illustrated the importance of vitamin D in maintaining cuff tendon health and tendon-to-bone healing in tendon tears. Since the production of 25-hydroxy (25-OH) vitamin D occurs in the liver, liver disease can induce a vitamin D-deficient condition and further impair cuff tendon health. We are the first to identify chronic liver disease as a risk factor for RCT, but further studies are necessary to clarify its underlying mechanism. 

Concerning other covariates, gout and hypertension were significantly correlated with RCT formation in the univariate, but not in the multivariate, Cox regression analysis. Hypertension was a significant risk factor for RCT in Gumina’s study [20], which claimed that hypovascularity resulting from hypertension predisposed tendons to tear from a very early stage. Compared to their case number of 408 with RCT, the case number in our study was much smaller. In addition to the case number discrepancy, different study designs and methodologies used in their study and ours also explained the different results. Huang et al. demonstrated that gout is a strong predisposing factor for RP [21]. They conducted a gout-and-control-cohort study comprising 98,169 patients using a national database from Taiwan over a 7-year follow-up period. The large sample size and different study designs made our results not comparable. We suggest that gout and hypertension contribute to pathologies in cuff tendons but are less prominent than smoking and chronic liver disease in our study.

The strength of this study is the use of a clinical database from a medical center that covers a large number of residents in southern Taiwan. We have a large sample size, a 1:4 matched control group, and a longer duration compared to a previous study [36]. Our study also has some limitations. First, we have no access to personal history and environmental factors, type, and severity of shoulder disease, the dose of steroids, and methods of injection from the clinical database. Second, we do not know the generalizability of our results from a medical center to a broader population or region. Further large-scale studies are needed to obtain more information regarding the adverse effects of steroid injections in shoulder diseases.

## 5. Conclusions

Steroid injections on the shoulder were associated with an increased risk of RCT by 7.44 times with a mean time to onset of 39 months compared to non-injection. The incidence of RCT was also elevated by 3.25 times when having concurrent chronic liver disease and by 2.4 times when having a smoking habit. We suggest that careful weighing of the benefits and adverse effects of steroids is necessary before administering injections for shoulder diseases.

## Figures and Tables

**Table 1:** Characteristics of the study population at the endpoint.

Variable	Total	With RCT	Without RCT	*p*-Value
	*n* = 1025 (%)	*n* = 30 (%)	*n* = 995 (%)	
Steroid injection	205 (20)	20 (67)	185 (19)	<0.001
Sex-Male	411 (40)	12 (40)	399 (40)	0.991
Age, years (Mean ± SD)	59.4 ± 12.5	62.2 ± 11.3	59.3 ± 12.5	0.200
Smoking	94 (9)	7 (23)	87 (9)	0.006
Alcohol use	64 (6)	4 (13)	60 (6)	0.103
Comorbidity				
Thyroid disorder	39 (4)	2 (7)	37 (4)	0.406
Diabetes	132 (13)	7 (23)	125 (13)	0.083
Gout	42 (4)	4 (13)	38 (4)	0.010
Depression	25 (2)	1 (3)	24 (2)	0.747
Hypertension	249 (24)	13 (43)	236 (24)	0.014
Ischemic heart disease	127 (12)	5 (17)	122 (12)	0.471
Chronic liver disease	78 (8)	7 (23)	71 (7)	0.001
Chronic kidney disease	62 (6)	2 (7)	60 (6)	0.885
Connective tissue disease	34 (3)	1 (3)	33 (3)	0.996
Osteoporosis	47 (5)	3 (10)	44 (4)	0.150

Mean ± SD = Mean ± standard deviation, RCT = rotator cuff tendon tear.

**Table 2:** Factors of outcomes after univariate Cox regression analysis.

Variables	Hazard Ratio (95%CI)	*p* Value
Steroid injection	8.50 (3.98–18.16)	<0.001
Sex-Male	0.92 (0.44–1.91)	0.817
Age ≥ 65 year	1.70 (0.83–3.48)	0.147
Smoking	3.00 (1.29–7.00)	0.011
Alcohol use	2.27 (0.79–6.50)	0.128
Comorbidity		
Thyroid disorder	2.07 (0.49–8.69)	0.322
Diabetes	1.94 (0.83–4.52)	0.125
Gout	3.63 (1.27–10.41)	0.016
Depression	1.42 (0.19–10.40)	0.732
Hypertension	2.49 (1.21–5.13)	0.013
Ischemic heart disease	1.53 (0.58–3.99)	0.389
Chronic liver disease	3.79 (1.63–8.84)	0.002
Chronic kidney disease	1.11 (0.27–4.68)	0.883
Connective tissue disease	1.14 (0.16–8.36)	0.899
Osteoporosis	2.30 (0.70–7.58)	0.172

CI = confidence interval.

**Table 3:** Factors of outcomes after univariate Cox regression analysis.

Variables	Adjusted Hazard Ratio (95% CI)	*p* Value
Steroid injection	7.44 (3.45–16.00)	<0.001
Smoking	2.40 (1.02–5.66)	0.046
Chronic liver disease	3.25 (1.38–7.63)	0.007

CI = confidence interval.

## Data Availability

The clinical database of Kaohsiung Veterans General Hospital (KSVGH) used to support the findings of this study is restricted by the Kaohsiung Veterans General Hospital Committee on Human Research (KSVGH20-CT-16) in order to protect patient privacy. Data are available from Research Center of Medical Informatics of Kaohsiung Veterans General Hospital, ksnhird@vghks.gov.tw for researchers who meet the criteria for access to confidential data.

## Supplementary Materials

The following are available online at https://www.mdpi.com/article/10.3390/ijerph19084520/s1, Table S1. ICD-9-CM, ICD-10-CM, procedure code, drug code.
