# Supplementary material for: A Positive Correlation between Steroid Injections and Cuff Tendon Tears: A Cohort Study Using a Clinical Database
Source: Int J Environ Res Public Health. 2022 Apr 8;19(8):4520. doi: 10.3390/ijerph19084520 (PMC9031762; doi:10.3390/ijerph19084520)
Supplement: Supplementary file 1 [file ijerph-19-04520-s001.zip › ijerph-1637533-supplementary.pdf]

Table S1. ICD-9-CM, ICD-10-CM, procedure code, drug code.

	ICD9		ICD10	
Inclusion	726	Adhesive capsulitis of shoulder	M65.81	Other synovitis and tenosynovitis, shoulder
	726.1	Rotator cuff diseases	M75.0	Adhesive capsulitis of shoulder
	726.2	Other lesions of shoulder	M75.2	Bicipital tendinitis
			M75.3	Calcific tendinitis of shoulder
			M75.4	Impingement syndrome of shoulder
			M75.5	Bursitis of shoulder
			M75.8	Other shoulder lesions
			M75.9	Shoulder lesion, unspecified
Exclusion	812	Fracture of humerus	S42.2-42.4, S42.9	Fracture of humerus
Outcome	727.61, 727.62	Complete rupture of rotator cuff	M75.1	Rotator cuff tear or rupture, not specified as traumatic
Procedure	39005C	Intraarticular injection		
	39018C	Tendon injection		
	39024B	Trigger point injection(one)		
	64121B, 64122B	Cuff tendon repair surgery		
Drug code	A011475209, A011475221, A011475229, A011476209, A011476221, A011476229, AC11475209, AC11475229, AC11476221, AC11476229	Triamcinolone		
